# Cholesterol and Sphingomyelin Uniquely Alter the Rate of Transthyretin Aggregation and Decrease the Toxicity of Amyloid Fibrils

**DOI:** 10.1021/acs.jpclett.3c02613

**Published:** 2023-11-30

**Authors:** Abid Ali, Kiryl Zhaliazka, Tianyi Dou, Aidan P. Holman, Dmitry Kurouski

**Affiliations:** † Department of Biochemistry and Biophysics, Texas A&M University, College Station, Texas 77843, United States; ‡ Department of Entomology, Texas A&M University, College Station, Texas 77843, United States; § Department of Biomedical Engineering, Texas A&M University, College Station, Texas 77843, United States

## Abstract

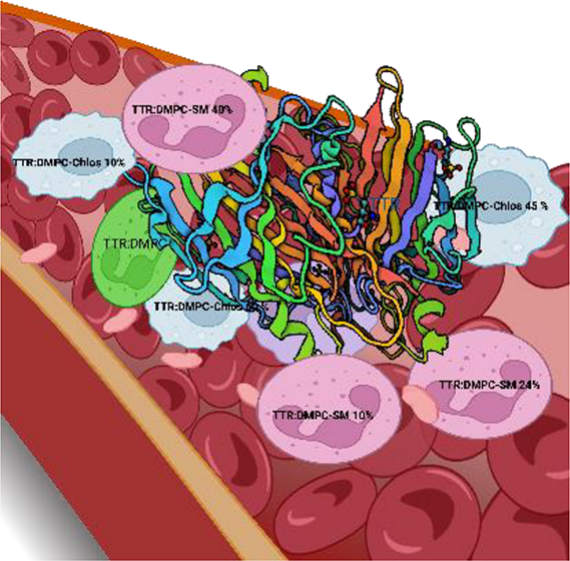

Transthyretin (TTR) is a small tetrameric protein that aggregates, forming highly toxic oligomers and fibrils. In the blood and cerebrospinal fluid, TTR can interact with various biomolecules, phospho- and sphingolipids, and cholesterol on the red blood cell plasma membrane. However, the role of these molecules in TTR aggregation remains unclear. In this study, we investigated the extent to which phosphatidylcholine (PC), sphingomyelin (SM), and cholesterol (Cho), important components of plasma membranes, could alter the rate of TTR aggregation. We found that PC and SM inhibited TTR aggregation whereas Cho strongly accelerated it. The presence of these lipids during the stage of protein aggregation uniquely altered the morphology and secondary structure of the TTR fibrils, which changed the toxicity of these protein aggregates. These results suggest that interactions of TTR with red blood cells, whose membranes are rich with these lipids, can trigger irreversible aggregation of TTR and cause transthyretin amyloidosis.

Transthyretin amyloidosis is a severe progressive pathology that impairs both central and peripheral neurons, as well as the heart and other organs.^1−4^ The disease is caused by abrupt aggregation or transthyretin (TTR), a small tetrameric protein that transports retinol and thyroid hormone thyroxine.^5−9^ As a result of protein aggregation, TTR forms highly toxic oligomers and fibrils that accumulate in organs and tissues.^10,11^ Biophysical analysis of TTR aggregation performed by the Kelly group demonstrated that unlike tetramers, TTR monomers were substantially less stable and could be easily misfolded at acidic pH.^12−14^ This triggered their irreversible assembly into oligomers and fibrils.^12−14^ Similar conclusions could be reached about a large number of amyloidogenic proteins, including insulin, lactalbumin, and lysozyme.^15−20^ These proteins lose their native fold at acidic pH, which triggers their assembly into amyloid oligomers and fibrils.^15−20^ Cryo-electron microscopy imaging of amyloid fibrils revealed that these aggregates possessed β-sheet-rich filaments.^21−24^ Such filaments were composed of two planes of β-sheets that stretched micrometers in length in the direction perpendicular to the peptide strands.

A growing body of evidence indicates that lipids can uniquely alter the aggregation rates of amyloidogenic proteins.^15,16,20,25−31^ For instance, Matveyenka and co-workers demonstrated that zwitterionic phosphatidylcholine (PC) could strongly inhibit insulin and lysozyme aggregation.^15,16,31^ At the same time, anionic lipids, such as phosphatidylserine (PS) and cardiolipin (CL), on the contrary, accelerated the aggregation of both proteins.^28,29^ It was also found that lipids not only altered the rates of protein aggregation but also uniquely modified the secondary structure of amyloid oligomers and fibrils formed in their presence.^15,16,30,31^ Such aggregates exerted drastically different cell toxicity compared to that of the oligomers and fibrils formed in the lipid-free environment.^32,33^ For instance, insulin oligomers formed in the presence of PC were found to be significantly less toxic than protein oligomers formed in the presence of PS and CL.^20,33^ However, amyloid fibrils formed by amyloid β_1–42_ in the presence of CL and cholesterol (Cho) exhibited much greater cell toxicity compared to that of amyloid β_1–42_ fibrils formed in the lipid-free environment.^34^ The results recently reported by Jakubec and co-workers showed that Cho strongly accelerated aggregation of α-synuclein (α-syn),^35^ a small protein that is linked to the onset and progression of Parkinson’s disease.^36^ This and other pieces of experimental evidence suggest that Cho can be an important player in the aggregation of amyloidogenic proteins.^34^ In addition, a study of the red blood cell plasma membrane reveals that Cho constitutes ∼40% of the plasma membrane lipids.^37^ Thus, one can expect that in the bloodstream misfolded TTR can interact with membranes of red blood cells, which can uniquely alter the toxicity of TTR oligomers and fibrils. Expanding upon this, we investigated the role of Cho in TTR aggregation. For this, we prepared unilamellar vesicles that contained 10%, 45%, and 60% Cho relative to 1,2-dimyristoyl-*sn*-glycero-3-phosphocholine (DMPC). These large unilamellar vesicles (LUVs) represent members with low, normal, and high concentrations of Cho, respectively.^37^ We also used a set of biophysical methods to determine the effect of different concentrations of Cho on the rate of TTR aggregation as well as the extent to which Cho can alter the morphology, secondary structure, and toxicity of TTR aggregates.

In addition to Cho and PC, 40–50% of the red blood cell plasma membrane is SM.^37^ Interestingly, neurons and red blood cells possess concentrations of SM much higher than those of other eukaryotic cells.^38^ In the membranes, SM is involved in many signaling pathways and in the determination of the membrane dynamics and fluidity.^38−41^ At the same time, the role of SM in the aggregation of misfolded proteins remains unclear. Matveyenka and co-workers found that SM decreased the rate of insulin aggregation.^15,16^ However, the opposite effect of SM was observed for lysozyme aggregation. Nevertheless, insulin:SM and lysozyme:SM fibrils were found to be less toxic than insulin aggregates formed in a lipid-free environment. Expanding upon this, we investigate the role of the SM in TTR aggregation. For this, we prepared LUVs that contained 10%, 24%, and 40% SM relative to PC. These lipid systems aim to model plasma membranes with low, normal, and high concentrations of SM, respectively. We applied the biophysical methods discussed above to unravel the extent to which SM could alter the rate of TTR aggregation, morphology, secondary structure, and toxicity of TTR aggregates.

We utilized a thioflavin T (ThT) assay to determine the effects of Cho and SM present at different molar concentrations with PC in LUVs on TTR aggregation. To understand the aggregation process, we focused on the duration of the lag phase (*t*_lag_, time required to reach 10% of the plateau phase intensity) and the half-time (*t*_1/2_, time required to reach 50% of the plateau phase intensity) TTR aggregation process.^42^ In the absence of LUVs, TTR aggregation exhibits a short lag phase (*t*_lag_ = 1.80 ± 0.07 h) that is followed by a rapid increase in the intensity of the ThT fluorescence signal (Figure 1). This increase is caused by the adsorption of ThT molecules to the β-strand structure in protein aggregates, which results in a change in the relative orientation of the aromatic rings in ThT. Thus, an increase in the ThT fluorescence can be used to monitor the rate of protein aggregation. We found that in the presence of LUVs of PC (100%) with a low concentration of Cho [Cho:PC (10:90)], the rate of TTR aggregation was decreased to 7.23 ± 1.02 and 7.96 ± 0.00 h, respectively (Figure 1). Thus, we can conclude that the presence of DMPC decelerated the TTR primary nucleation step. We also found that the TTR aggregation rate did not change (*t*_lag_ = 2.1 ± 0.2 h) in the presence of the medium [Cho:PC (45:55)] concentration of Cho compared to the rate of protein aggregation in the lipid-free environment (Figure 1). However, at the same PC concentration, an increase in the concentration of Cho from 45% to 60% [Cho:PC (60:40)] resulted in a drastic increase in the rate of TTR aggregation (Figure 1). These results demonstrated that at the higher concentration of Cho, TTR could nearly instantaneously aggregate, forming toxic protein aggregates. Thus, one can expect that an increase in the concentration of Cho in plasma membranes can trigger the abrupt aggregation of TTR.

**Figure 1:**
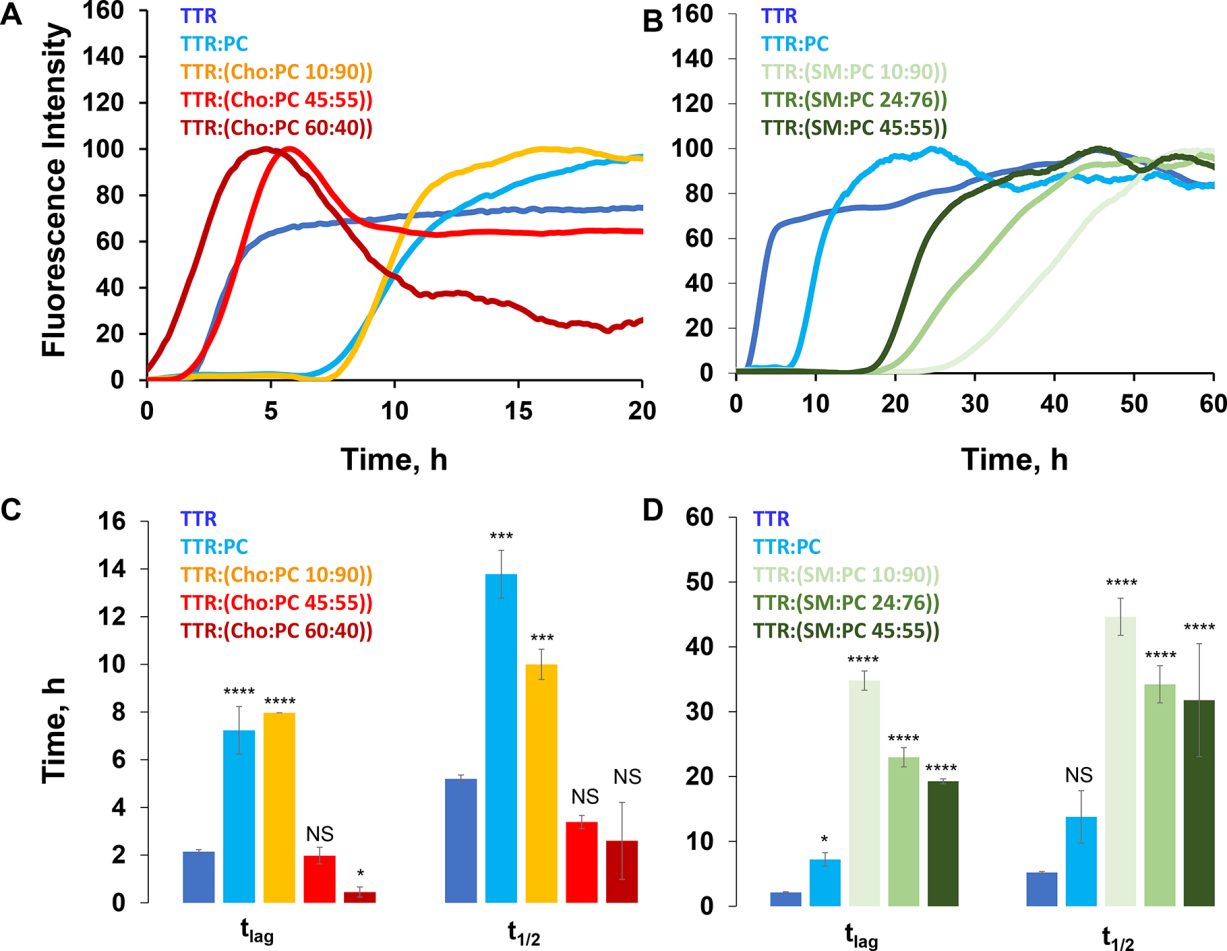
ThT aggregation kinetics of TTR in the lipid-free environment and in the presence of (A) PC, Cho:PC (10:90), Cho:PC (45:55), and Cho:PC (60:40) and (B) SM:PC (10:90), SM:PC (24:76), and SM:PC (45:55). Each kinetic curve is the average of three independent measurements. Corresponding bar graphs (C and D) show *t*_lag_ and *t*_1/2_, which correspond to 10% and 50% ThT intensity, respectively. Analysis of variance was used to determine the statistical significance in *t*_lag_ and *t*_1/2_. *P < 0.05. **P < 0.01. ***P < 0.001. ****P < 0.0001. NS indicates a nonsignificant difference.

Our results also demonstrated that the increased concentration of Cho reduced the lag phase but did not have a significant effect on the rate of TTR aggregation. Specifically, the *t*_1/2_ of TTR:Cho:PC (60:40) was 2.59 ± 1.61 h, whereas the *t*_1/2_ values of TTR:Cho:PC (45:55) and TTR were 3.38 ± 0.27 and 2.65 ± 0.15 h, respectively (Figure 1). At the same time, low concentrations of Cho have very little if any effect on the rate of TTR aggregation compared to the rate of protein aggregation in the presence of PC LUVs alone.

The ThT results demonstrate that SM at low concentrations (10%) strongly inhibited (*t*_lag_ = 34.79 ± 1.48 h) TTR aggregation. We also observed a progressive decrease in the inhibition effect of SM as its concentration increased. Specifically, *t*_lag_ values of 22.97 ± 0.2 and 19.27 ± 0.36 h were determined in the presence of SM:PC (24:76) and SM:PC (40:60), respectively (Figure 1). It should be noted that the SM-driven inhibition of TTR aggregation was much stronger than the inhibition exerted by PC itself. Similar conclusions could be reached about the effect of SM on the rate of TTR aggregation. We found that the *t*_1/2_ of TTR aggregation was decreased to 44.63 ± 2.86 h in the presence of SM:PC (10:90), whereas SM:PC (24:76) and SM:PC (40:60) exerted the same *t*_1/2_ equal to 34.22 ± 2.86 and 31.78 ± 8.70 h, respectively (Figure 1). These results demonstrated that SM strongly inhibited TTR aggregation. Furthermore, the inhibition effect is inversely proportional to the concentration of SM in LUVs.

Using atomic force microscopy (AFM), we investigated the morphology of TTR aggregates grown in the presence of Cho and SM at different molar ratios relative to PC, as well as in the lipid-free environment (Figure 2 and Figure S1). Our results demonstrated that in the lipid-free environment, TTR formed thin and short fibrils. These aggregates were 5–9 nm in height. In the presence of PC, TTR aggregated primarily, forming oligomers, spherical specimens that were 4–8 nm in height (Figure 2 and Figure S1). We also observed the presence of thick fibril bundles, suggesting that lipids facilitate the assembly of thin fibrils that were also observed in TTR:PC into large supramolecular assemblies. Similar thin fibrils and their supramolecular assemblies were observed in TTR:[SM:PC (10:90)]. We observed morphologically similar fibrils in TTR:[SM:PC (24:76)] and TTR:[SM:PC (40:60)]. Although fibrils formed in SM:PC (24:76) LUVs exhibited a similar thickness, an increase in the concentration of SM relative to PC (40:60) resulted in a formation of much thicker fibrils (medium from 7.5 to 10.5 nm). These results demonstrate that high concentrations of SM promote fibrilization of TTR into thicker fibrils. Specifically, TTR:[SM:PC (40:60)] fibrils had nearly 2 times larger heights compared to the heights of those formed by TTR in the lipid-free environment. It should be noted that we did not observe lipid LUVs, which indicates that TTR, like α-synuclein, interacted with these vesicles during the early stages of protein aggregation.^43^

**Figure 2:**
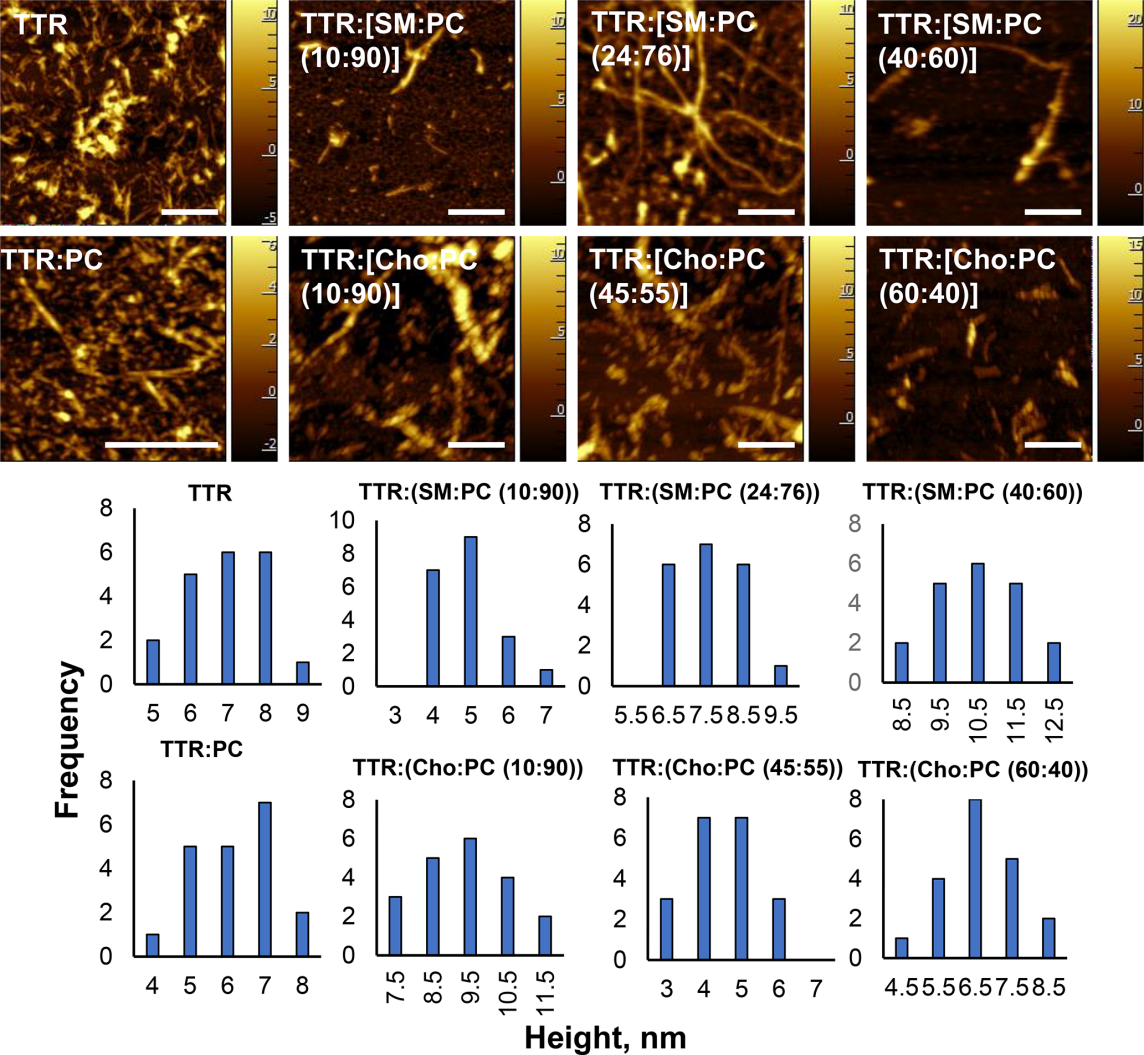
AFM images (top) of TTR aggregates grown in the lipid-free environment and PC and in the presence of SM and Cho at different molar ratios relative to PC. All images correspond to protein aggregation for 48 h at 37 °C. The scale bar is 500 nm. Histograms (bottom) of the height distribution of the observed aggregates. On average, 25 individual protein aggregates were measured in each sample.

AFM imaging revealed that in the presence of low, medium, and high concentrations of Cho, TTR primarily formed oligomers similar to those observed in TTR:PC. We also found that some of those aggregates assembled side by side forming fibrillar structures. AFM did not reveal drastic changes in the morphology of these aggregates as the concentration of Cho increased relative to the concentration of PC. We found that TTR:[Cho:PC (10:90)] aggregates had only slightly larger heights compared to those of TTR:[Cho:PC (45:55)] and TTR:[Cho:PC (60:40)]. These results demonstrated that PC and SM uniquely altered the morphologies of protein aggregates. However, the effect of Cho is rather weak or indistinguishable from the effect of PC on the morphology of the TTR aggregates.

We used Fourier transform infrared (FTIR) spectroscopy to probe the secondary structure of TTR:PC, TTR:SM, and TTR:Cho samples as well as TTR aggregates formed in the lipid-free environment (Figure 3). In the acquired IR spectra, we observed amide II (1525–1555 cm^–1^) and an amide I band centered at ∼1632 cm^–1^.^28^ The position of the amide I band suggests that all analyzed aggregates possessed a large amount of parallel β-sheet.^44,45^ However, it should be noted that FTIR probes the bulk volume of the protein samples. We also think that the presence of unaggregated protein largely affects the readout of FTIR. Thus, this technique cannot be used to resolve the secondary structure of individual fibrils.^44^ To overcome this limitation, we utilized nano-infrared spectroscopy, also known as atomic force microscopy infrared spectroscopy (AFM-IR).^46,47^ In AFM-IR, a metalized scanning probe is positioned directly at the protein aggregate.^44,48,49^ Next, the sample is illuminated by pulsed tunable IR radiation, which causes thermal expansions in the sample of interest.^50−52^ These thermal expansions are transferred to the scanning probe and converted into the IR spectra, which correspond to the structure of the analyzed object of interest.^53−55^

**Figure 3:**
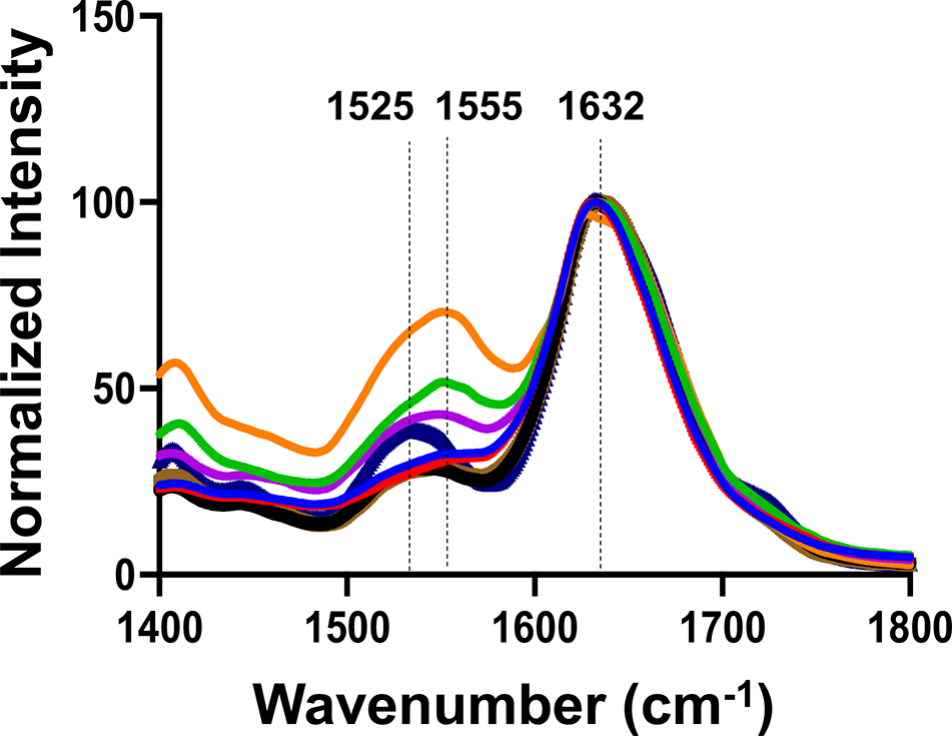
FTIR spectra of TTR (blue), TTR:PC (red), TTR:[Cho:PC (10:90)] (green), TTR:[Cho:PC (45:55)] (purple), TTR:[Cho:PC (60:40)] (orange), TTR:[SM:PC (10:90)] (black), TTR:[SM:PC (24:76)] (brown), and TTR:[SM:PC (40:60)] (marine).

In the acquired AFM-IR spectra from TTR fibrils, we observed the amide I band centered around 1632–1674 cm^–1^ (Figure 4 and Figure S2). The fitting of amide I revealed that the secondary structure of TTR fibrils was dominated by parallel β-sheet (1624 cm^–1^) with a substantial amount of α-helix and random coil (1655 cm^–1^) and antiparallel β-sheet (1694 cm^–1^) present in these aggregates (Figures S3 and S4). We found that the secondary structures of TTR:[Cho:PC (15:85)] and TTR:PC were very similar. In both cases, we observed nearly identical amounts of parallel β-sheets and α-helix/random coil. We also found that with an increase in the concentration of Cho relative to that of PC, the amount of α-helix/random coil was increasing in TTR aggregates with a graduate decrease in the amount of both parallel and antiparallel β-sheet. These results demonstrated that both PC and Cho uniquely altered the secondary structure of the TTR aggregates. It should be noted that in the acquired AFM-IR spectra from TTR aggregates grown in the presence of lipids, we observed the vibrational band centered ∼1730 cm^–1^. This vibrational band originates from the carbonyl (C=O) vibration of ester groups present in phospholipids.^32^ On the basis of this result, we can conclude that PC was present in the structure of TTR fibrils that were formed in the presence of PC and Cho. It should be noted that the IR spectrum of Cho has two intense vibrational bands at 1470 and 1055 cm^–1^ that are also present in the IR spectrum of PC (Figure S5). Therefore, we cannot unambiguously confirm the presence or absence of Cho in TTR fibrils that were grown at different Cho:PC ratios. The same conclusion could be reached about TTR fibrils grown in the presence of SM. Specifically, SM does not have a carbonyl vibration at 1730 cm^–1^. At the same time, the IR spectrum of this lipid does not have a unique marker band that can be used to identify the presence of SM in TTR fibrils. Therefore, we cannot unambiguously confirm the presence or absence of SM in TTR fibrils that were grown at different SM:PC ratios.

**Figure 4:**
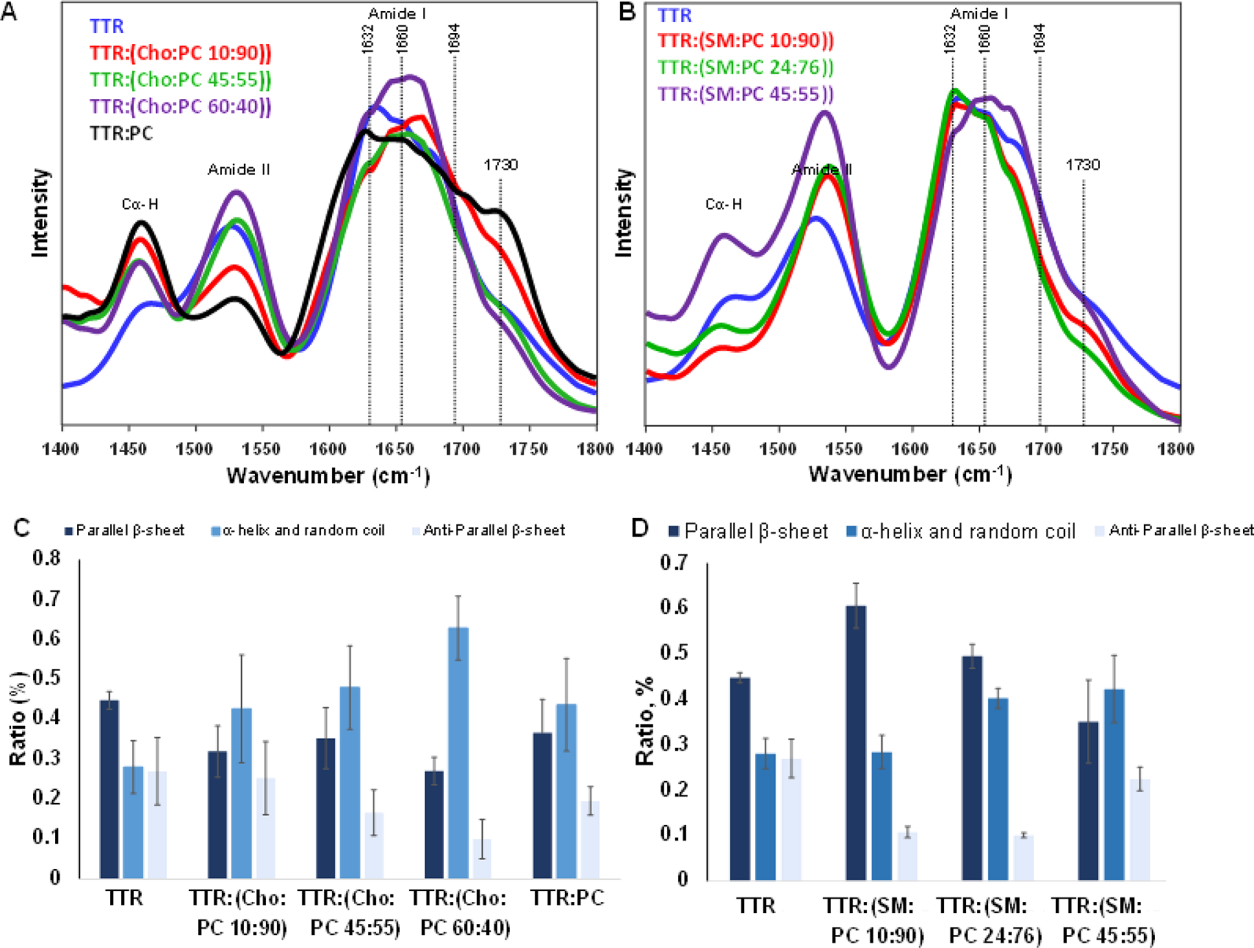
Averaged AFM-IR spectra acquired from (A) TTR fibrils formed in the presence of 10:90, 45:55, and 60:40 Cho:PC and 100% PC and in the lipid-free environment (TTR) and (B) TTR fibrils formed in the presence of 10:90, 24:76, and 45:55 SM:PC. (C and D) Corresponding bar graphs summarize the distribution of protein secondary structure in the protein aggregates according to the fitting of the amide I band: dark blue for parallel β-sheet (1632 cm^–1^), blue for α-helix and random coil (1660 cm^–1^), and light blue for antiparallel β-sheet (1674 cm^–1^).

AFM-IR analysis revealed a gradual decrease in the amount of parallel β-sheet in TTR aggregates formed in the presence of SM:PC as the ratio of SM increased from 10% to 60% relative to the ratio of PC in LUVs. We also observed a gradual increase in the amount of antiparallel β-sheet and α-helix/random coil in these aggregates. These results demonstrated that the concentration of SM in the LUVs alters the protein secondary structures in TTR fibrils and the increase in fibril thickness may be related to the increase in the level of antiparallel β-sheet conformation. It should be noted that an increase in the amount of the α-helix/random coil in TTR aggregates formed in the presence of SM:PC LUVs could be explained by an increase in the concentration of SM in the structure of such fibrils. SM has a vibrational band at 1655 cm^–1^ (Figure S5), which overlaps with the amide I band of proteins, where it can be assigned to α-helix/random coil secondary structure. Consequently, if the concentration of SM in such protein aggregates increases from 10% to 45% relative to the concentration of PC, the intensity of 1655 cm^–1^ will also increase. However, this increase is not due to the changes in the secondary structure in these aggregates but rather to the increase in the concentration of SM. Therefore, due to the presence of the feature at 1655 cm^–1^ in the IR spectrum of SM, IR cannot be used to reach ubiquitous conclusions about changes in the α-helix/random coil secondary structure of TTR aggregates formed in the presence of SM:PC LUVs.

We investigated whether the structural differences in TTR aggregates discussed above formed in the presence of PC, SM, and Cho, as well as fibrils grown in the lipid-free environment, had similar or different cell toxicity. For this, protein aggregates formed after TTR agitation for 48 h at 510 rpm and 37 °C were exposed to N27 ran dopaminergic cells. After the cells had been exposed to the aggregates for 24 h, the LDH assay was performed to investigate the extent to which TTR aggregates were toxic to N27 cells. The results of the LDH assay demonstrated that TTR fibrils were far more toxic than TTR:PC fibrils. We also found that all TTR:(PC:Cho) fibrils exerted similar cell toxicity compared to that of TTR:PC aggregates (Figure 5). LDH also revealed that TTR:(PC:SM) fibrils were not toxic to N27 ran dopaminergic cells, whereas TTR:[PC:SM (40:60)] had some neuroprotective effect (Figure 5). It should be noted that lipids themselves were found to be nontoxic to the cells (Figure S6). On the basis of these results, we can conclude that PC and SM uniquely alter the toxicity of TTR aggregates. Specifically, the presence of SM in LUVs drastically reduced the cytotoxicity of TTR fibrils compared with that of the aggregates formed by TTR in the lipid-free environment. Although the same conclusions could be reached about PC, this lipid causes a significantly smaller decrease in the toxicity of TTR aggregates formed in its presence. Finally, our results demonstrated that Cho has very little, if any, effect on the toxicity of TTR fibrils formed in its presence at 15–60% relative to the concentration of PC in LUVs.

**Figure 5:**
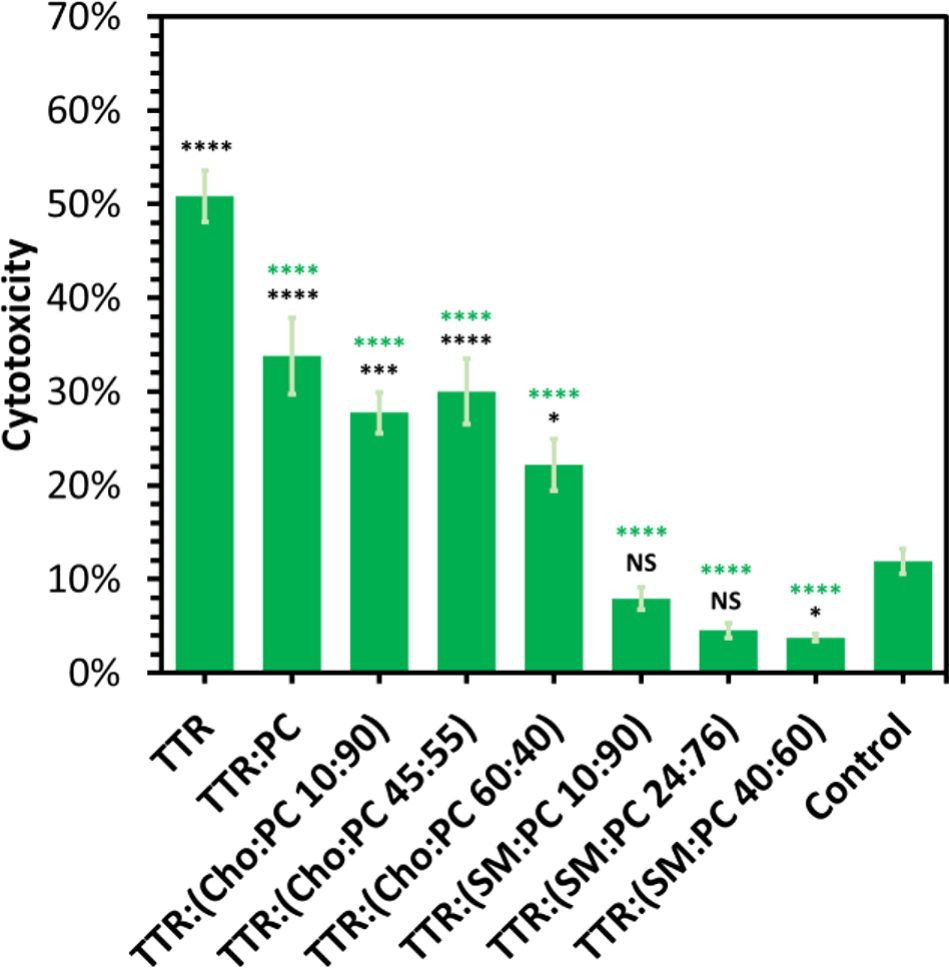
Toxicity of TTR aggregates grown in the presence of lipids determined by the chemical structure of the lipid. Histograms of LDH (top), ROS (middle), and JC-1 (bottom) toxicity assays of Ins, Ins:PC, Ins:PE, Ins:PG, Ins:PS, and Ins:CL, as well as PC, PE, PG, PS, and CL.

Our results showed that the rate of TTR aggregation could be uniquely altered by lipids. Specifically, PC and SM strongly decelerated aggregation, whereas Cho facilitated TTR aggregation. Similar findings were reported by Matveyenka and co-workers.^15,16^ Specifically, the researchers demonstrated that both PC and SM strongly inhibited insulin aggregation. Furthermore, Zhaliazka and co-workers found that this effect could be attributed to all zwitterionic lipids.^20^ Our results are also consistent with the experimental findings reported by Jakubec and co-workers according to which Cho strongly accelerated the aggregation of α-syn.^35^

Our study also revealed the extent to which the rate of TTR aggregation could be directly dependent on the amounts of SM and Cho in PC LUVs. We found that with a decrease in the concentration of SM relative to PC from 10% to 60%, an increase in the inhibition activity of SM was observed. Similar results were also found for Cho. We observed a gradual increase in the rate of TTR aggregation as the concentration of Cho increased from 10% to 60%. It should be noted that TTR nearly instantaneously formed fibrils at the higher concentration of Cho (60%). These results suggest that a change in the lipid profile of plasma membranes, specifically an increase in the concentration of Cho, may trigger TTR aggregation.

We also found that Cho, SM, and PC not only altered the rate of TTR aggregation but also uniquely changed the morphology of TTR fibrils. Specifically, in a lipid-free environment, TTR primarily formed thin and short fibrils. We found that these fibrils could form higher supramolecular assemblies in the presence of SM. Finally, the presence of PC oligomers rather than fibrils was primarily observed. It should be noted that we did not observe any significant impact of Cho on the morphology of the TTR fibrils that were formed in the presence of this lipid.

AFM-IR analysis of protein aggregates showed that lipids could uniquely alter the secondary structure of the TTR fibrils. These structural differences had a significant impact on the toxicity that TTR aggregates exerted on N27 cells. We found that in the presence of PC, SM, and Cho, TTR formed aggregates that exerted significantly less cell toxicity compared to the effect of those formed in the lipid-free environment. These results are consistent with the experimental findings that were reported by Matveyenka and co-workers for insulin.^15,16,29^ The researchers demonstrated that insulin:PC and insulin:SM fibrils were significantly less toxic to N27 cells than insulin aggregates formed in the lipid-free environment.^15,16^ Similar to our current findings, Rizevsky and co-workers discovered that such aggregates possessed lipids in their structure.^32^ One could expect that the changes in the aggregate toxicity discussed above could be determined by the presence of lipids in their structure. Such lipids could alter the propensity of protein aggregates to cross lipid bilayers and damage endosomes and mitochondria. Alternatively, the drastically low toxicity of TTR:SM fibrils could be explained by the substantially lower likelihood of such long aggregates entering the cell.^11^ Our previously reported results demonstrated that amyloid fibrils are endocytosed by cells.^15^ Consequently, endocytosis of small and thick fibrils, as well as oligomers, is more feasible than that of long thick fibril bundles that were formed in the presence of SM. A similar hypothesis was proposed by Ramamoorthy’s group based on the analysis of cytotoxicity of Aβ_1–42_ aggregates grown in the presence of 1,2-dilauroyl-*sn*-glycero-3-phosphatidylcholine (DLPC).^56^ The researchers observed that the resulting fibrils were significantly less toxic than Aβ_1–42_ aggregates formed in a DLPC-free environment. On the basis of these results, Ramamoorthy’s group hypothesized that zwitterionic LUVs can be used as therapeutic platform to decrease the toxicity of amyloid fibrils. Our recent findings fully support this hypothesis.

In summary, our findings demonstrate that lipids can play an important role in TTR amyloidosis. Specifically, SM-, Cho-, and PC-rich membranes of red blood cells can uniquely alter the stability of TTR by decelerating or accelerating its aggregation. This effect is determined not only by the chemical structure of the lipid but also by its concentration in the lipid bilayer in such membranes. These results suggest that TTR amyloidosis could be linked not only to TTR misfolding but also to the change in the lipid profile of red blood and epithelial cells that can come in contact with such misfolded proteins.

## Supporting Information Available

Additional experimental details, materials, and methods; AFM images of the analyzed samples (Figures S1 and S2); FTIR spectra acquired from PC and SM (Figure S3); and cytotoxicity results of lipid samples used in the study (Figure S4) (PDF)
