# Supplementary material for: Cholesterol and Sphingomyelin Uniquely Alter the Rate of Transthyretin Aggregation and Decrease the Toxicity of Amyloid Fibrils
Source: J Phys Chem Lett. 2023 Nov 30;14(49):10886–93. doi: 10.1021/acs.jpclett.3c02613 (PMC10863059; doi:10.1021/acs.jpclett.3c02613)
Supplement: Supplementary file 1 — jz3c02613_si_001.pdf [file jz3c02613_si_001.pdf]

# Cholesterol and Sphingomyelin Uniquely Alter the Rate of Transthyretin Aggregation and Lower the Toxicity of Amyloid Fibrils

Abid Ali[1], Kiryl Zhaliazka[1], Tianyi Dou[1], Aidan P. Holman[2] and Dmitry Kurouski[1,3*]

1. Department of Biochemistry and Biophysics, Texas A&M University, College Station, Texas 77843, United States
2. Department of Entomology, Texas A&M University, College Station, Texas 77843, United States
3. Department of Biomedical Engineering, Texas A&M University, College Station, Texas, 77843, United States

Email: dkurouski@tamu.edu

# Supporting Information

**Materials:** 1,2-Dimyristoyl-sn-glycero-3-phosphocholine (DMPC or PC), sphingomyelin and cholesterol were purchased from Avanti (Alabaster, AL, USA). TTR was expressed in the lab in *Escherichia coli* BL21 (DE3) strain using LB broth media according to the protocol described by Volles and Lansbury.[1, 2]

**Liposome preparation:** LUVs of PC, SM and Cho were prepared according to the procedure reported by Mateyenka and co-workers [3-5]. The lipid weights were calculated based on their ratio. For DMPC, 400 µM were used to make PC: Cho and SM mixtures. Briefly, lipids were first dissolved in chloroform; after the solvent was evaporated, lipid film was re-dissolved in PBS, pH 7.4. Next, a heating-thawing cycle was performed to the lipid solutions. For this, solutions were first heated to 50°C for 30 min and the immersed into lipid nitrogen for 3-5 min. Finally, lipid solutions were exposed to the extruding procedure in which the solutions were passed 20-30 times through 100 nm membrane (Avanti, Alabaster, AL, USA). LUV sizes were determined by dynamic light scattering. All samples had LUV sizes of 100 ±10 nm.

**Protein aggregation and kinetic measurements:** 50 µM of TTR was dissolved in 0.1M sodium acetate buffer that contained 1M KCl; pH 3.0. For protein:lipid samples, 50 µM of TTR was mixed with an equivalent concentration of the corresponding LUVs. Solution pH was adjusted to pH 3.0. All samples were placed into a well-plate that was agitated for 20-60 h with 510 rpm, 37 °C (Tecan, Mannedorf, Switzerland). For kinetic measurements, ThT was added to the sample to reach the final concentration of 25 µM. Samples

were incubated at the same experimental conditions using the same equipment (Tecan, Mannedorf, Switzerland). Fluorescence measurements were taken every 10 min; excitation was 450 nm; emission was collected at 488 nm.

**AFM imaging:** AFM images were collected using AIST-NT-HORIBA system (Edison, NJ) in tapping mode. For each measurement, an aliquot of samples incubated at 37 °C for 48h was diluted with PBS and then deposited onto a pre-cleaned glass coverslip. The same was left drying on the glass coverslip surface (Ted Pella, Redding, CA). After the sample was fully dried, the glass surface was rinsed by DI water and dried under a flow of dry nitrogen. Tapping mode gold-coated AFM probes (force constant 2.7 N/m and resonance frequency 50–80 kHz) were used for AFM imaging (Appnano, Mountain View, CA, USA)). AIST-NT image processing software was used to analyze AFM images. The following image processing steps were made: Facet leveling; Iterative Polynomial Background Leveling; Fit Line Correction; Remove Scar Line Correction.

**AFM-IR:** AFM-IR spectra were acquired with 4 $cm^{-1}$ spectral resolution using the Nano-IR3 system (Bruker, Santa Barbara, CA, USA) equipped with contact-mode gold-coated AFM tips (ContGB-G AFM probes, NanoAndMore). The scanning probes had 0.2 N/m force constant with 13 kHz resonance frequency. Scan rate was 1Hz with a setpoint ranging from -0.73 to -0.9V. Data analysis was performed in Matlab (The Mathworks, Inc. Natick, Massachusetts, USA). Experimentally, we found that amyloid fibrils very quickly adhered to the surfaces of silicon (Si) wafers (Ted Pella, Redding, CA). Therefore, we exposed the suspension of fibrils on the surface of Si wafers only for a few minutes. Next, the surface was rinsed by DI water and dried under a flow of dry nitrogen.

**AFM-IR spectral deconvolution:** The spectra collected from each aggregate were baselined and averaged in Matlab. The processed spectra were input into the GRAMS/AI for peak fitting. The parallel β-sheet, α-helix and random coil, and anti-parallel β-sheet were fitted around 1624, 1655, and 1694 $cm^{-1}$. The percentages of each amide I structure were calculated based on the fitted areas and the total amide I area. The error bars represent both variability in spectra acquired from different protein aggregates in the same sample and errors in fitting.

**Fourier-Transformed Infrared Spectroscopy (FTIR):** Protein samples were placed onto ATR crystal and dried at room temperature. Spectra were measured with 4 $cm^{-1}$ resolution using Spectrum 100 FTIR spectrometer (Perkin-Elmer, Waltham, MA, USA). Three spectra were collected from each sample.

**Cell toxicity assays:** The N27 rat dopaminergic neuron cell line was purchased from Sigma-Aldrich (St. Louis, MO). Cells were cultured in 96-well plates with RPMI 1640 Medium supplemented with 10% fetal bovine serum (FBS) at 37°C with 5% $CO_2$. After 24 hours cells were found fully adherent. For the LDH assay, 100 μL of the cell culture was replaced with 100 μL of RPMI 1640 Medium containing 5% FBS and 10 μL of the protein samples. After 24 hours of incubation, the amount of lactate dehydrogenase (LDH) released into the cell culture medium was measured using the non-radioactive CytoTox 96 cytotoxicity assay kit (G1781, Promega, Madison, WI, USA). The toxicity of the protein aggregates towards N27 cells was measured by the level of formazan produced, which directly correlated with the amount of LDH released using absorbance read at 490 nm.

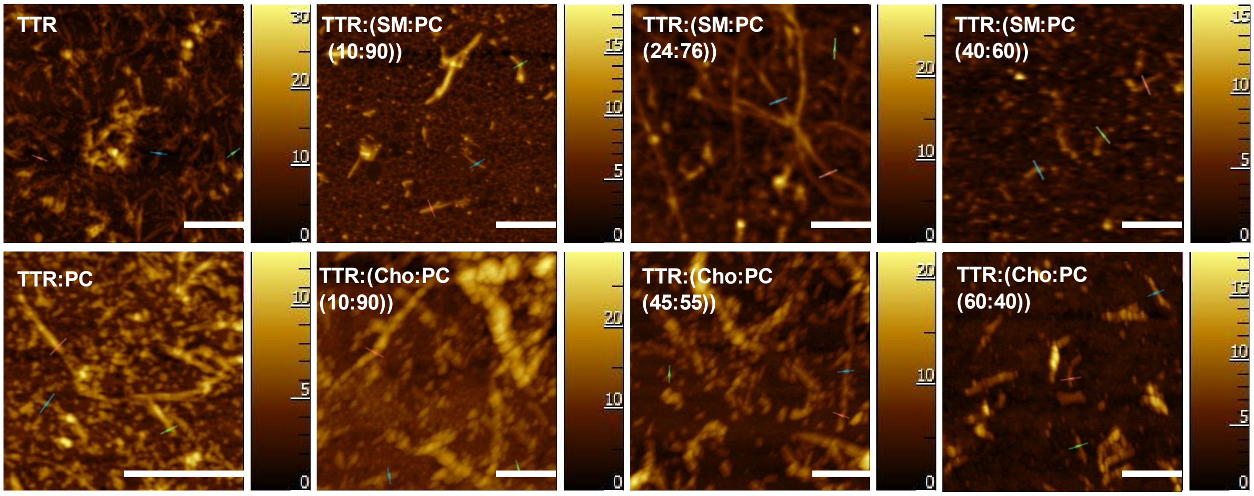

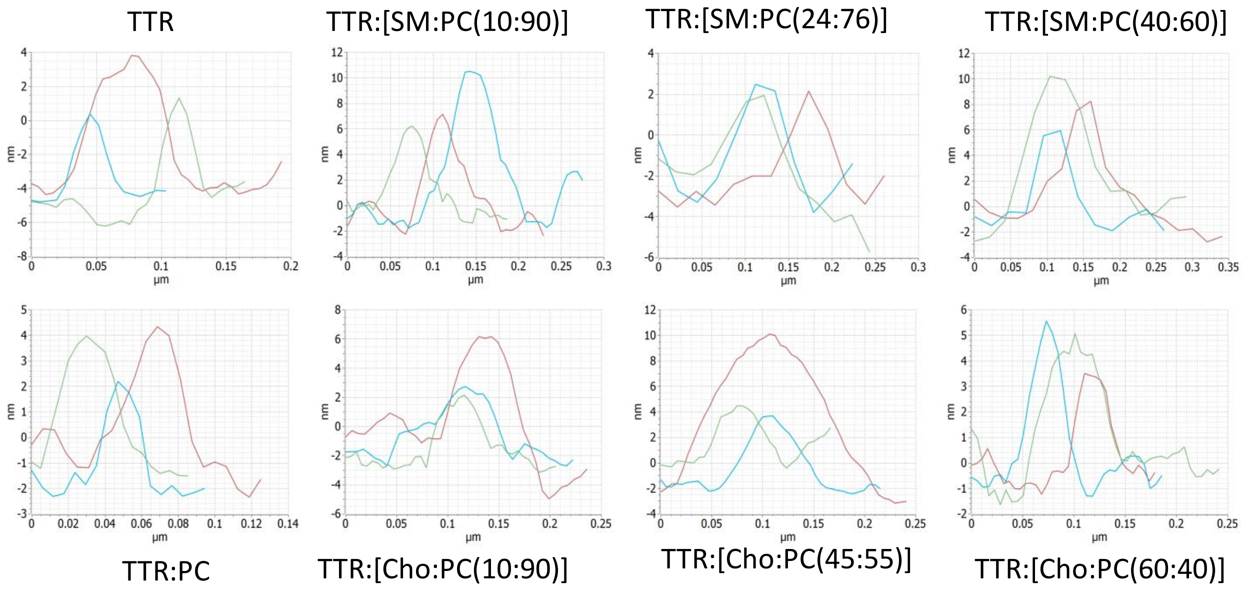


Figure S1. AFM images (top) with the corresponding height profiles (bottom). Scale bars are 500 nm.

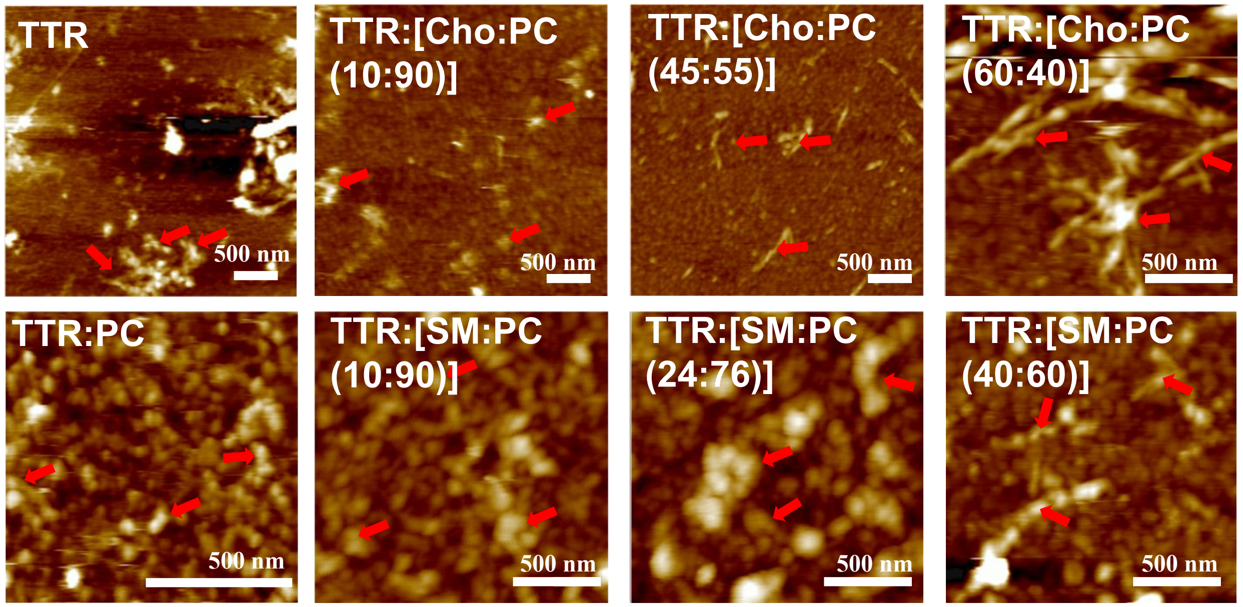


**Figure S2.** AFM images with sites from which AFM-IR spectra were acquired (showed by red arrows).

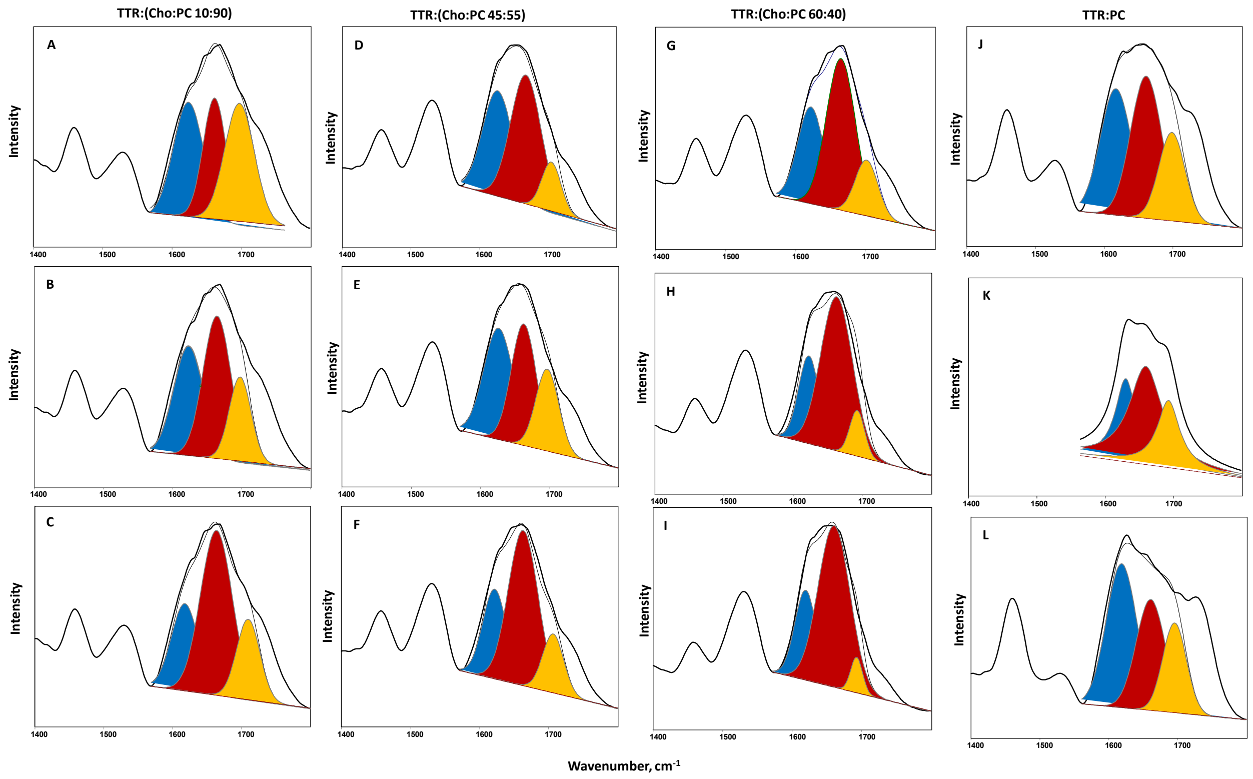


**Figure S3.** The AFM-IR deconvolution spectra of TTR aggregates formed in the presence of Cho:PC (10:90) (A-C), Cho:PC (45:55) (D-F), Cho:PC (60:40) (G-I) and PC (J-L). The parallel β-sheet is in blue, the α-helix and unordered protein is in blue, and the anti-parallel β-sheet is in yellow.

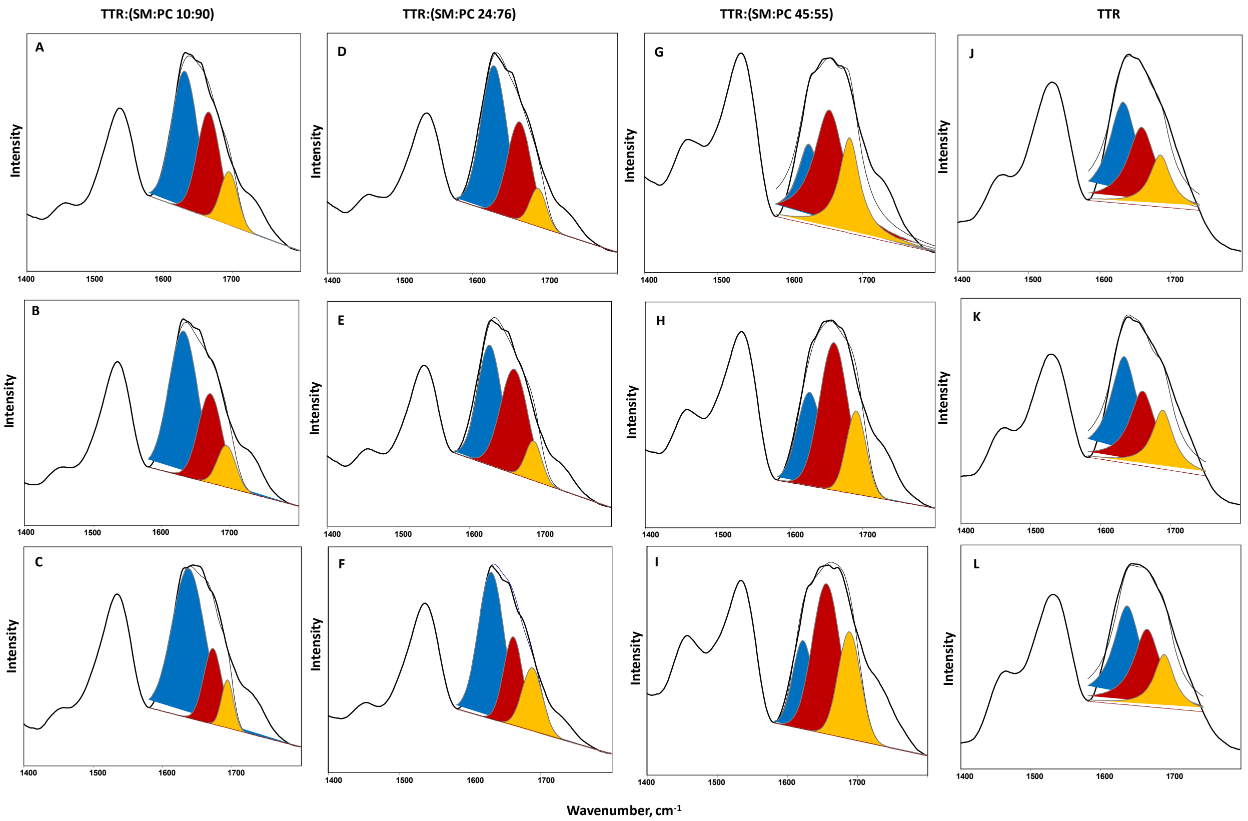


**Figure S4.** The AFM-IR deconvolution spectra of TTR aggregates formed in the presence of SM:PC (10:90) (A-C), SM:PC (24:76) (D-F), SM:PC (45:55) (G-I) and TTR itself (J-L). The parallel β-sheet is in blue, the α-helix and unordered protein is in blue, and the anti-parallel β-sheet is in yellow.

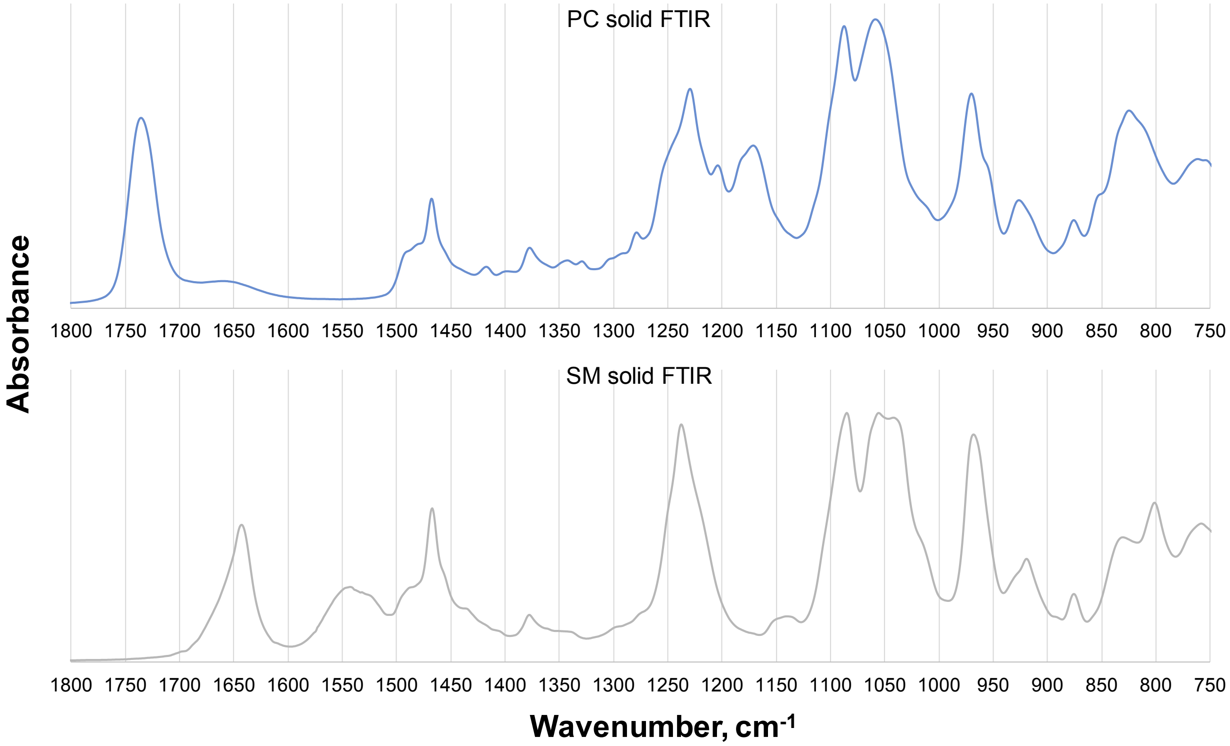


**Figure S5.** FTIR spectra of solid PC and SM.

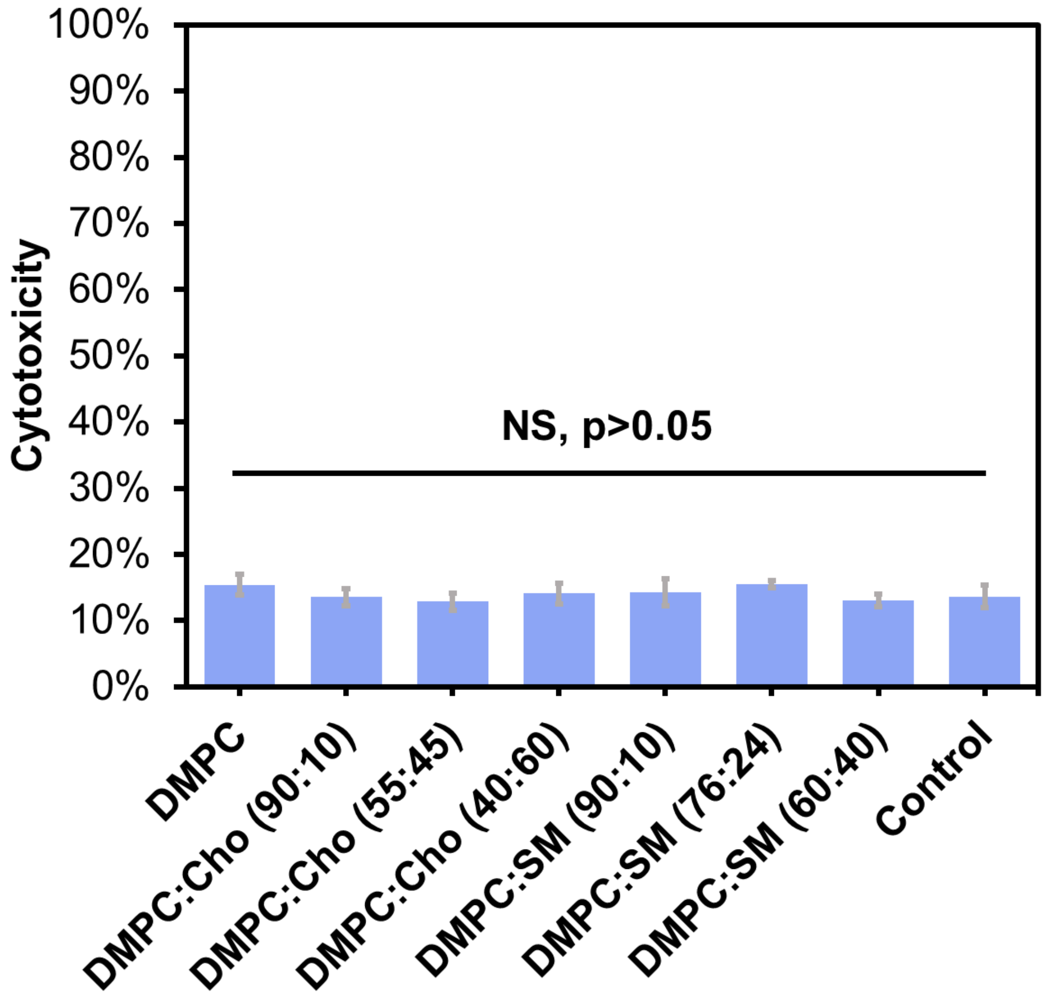


**Figure S6.** The toxicity of lipids themselves DMPC and mixer of the DMPC: Cho(90:10), DMPC: Cho(55:45), DMPC: Cho(40:60), DMPC: SM(90:10), DMPC: SM(76:24), DMPC: SM(60:40),

**References:**

1. Singh, P. K.; Kotia, V.; Ghosh, D.; Mohite, G. M.; Kumar, A.; Maji, S. K., Curcumin modulates alpha-synuclein aggregation and toxicity. *ACS Chem. Neurosci.* **2013,** *4* (3), 393-407.

2. Volles, M. J.; Lansbury, P. T., Jr., Relationships between the sequence of alpha-synuclein and its membrane affinity, fibrillization propensity, and yeast toxicity. *J. Mol. Biol.* **2007,** *366* (5), 1510-22.

3. Matveyenka, M.; Rizevsky, S.; Kurouski, D., Amyloid aggregates exert cell toxicity causing irreversible damages in the endoplasmic reticulum. *Biochim. Biophys. Acta Mol. Basis Dis.* **2022,** *1868* (11), 166485.

4. Matveyenka, M.; Rizevsky, S.; Pellois, J. P.; Kurouski, D., Lipids uniquely alter rates of insulin aggregation and lower toxicity of amyloid aggregates. *Biochim. Biophys. Acta Mol. Cell. Biol. Lipids* **2023,** *1868* (1), 159247.

5. Matveyenka, M.; Zhaliazka, K.; Rizevsky, S.; Kurouski, D., Lipids uniquely alter secondary structure and toxicity of lysozyme aggregates. *FASEB J.* **2022,** *36* (10), e22543.
